# Maintenance and repair of an aging life cycle

**DOI:** 10.18632/oncotarget.18046

**Published:** 2017-05-21

**Authors:** Marjolein P. Baar, Hester Van Willigenburg, Peter L.J. de Keizer

**Affiliations:** Department of Molecular Genetics, Erasmus University Medical Center, Wytemaweg, Rotterdam, The Netherlands

**Keywords:** senescence, aging, apoptosis, FOXO, P53

“Targeting signs of aging”. It sounds more like a punch-line of a TV commercial, than a consequence of fundamental science. But as we observed recently, it might actually be possible to achieve just that, using a prospectively designed FOXO4-p53 interfering peptide that targets so-called “senescent” cells [1]. More research is needed to fully assess its true translational potential and whether it is even safe to remove such cells. However, these findings pose a very attractive starting point to develop ways to live out our final years in better health.

Aging has often been considered as an integral part of life; a form of “noise” that cannot be targeted or tampered with. This is in part because for long the underlying causes of organismal aging were simply too elusive to comprehend, let alone modify. The chronic build-up of DNA damage has now evidently been established as a major cause for aging, but to counteract the genomic damage that has occurred over a lifetime is an entirely different challenge altogether [2]. One approach to overcome this issue, is to eliminate those cells that are too damaged to faithfully perform their duty and to replace them by fresh and healthy counterparts. Senescent cells are exciting candidates for such an approach. Comparable to formation of rust on old equipment, like a bicycle (Figure 1), they accumulate during aging and especially at sites of pathology. They develop a chronic secretory profile that is thought to impair tissue renewal and contribute to disease development, for instance by keeping neighboring cells “locked” in a permanent state of stemness [2]. Senescence can be beneficial in a transient setting, but the genetic removal of senescent cells over a prolonged period of time was found to be safe and to potently extended health- and lifespan of naturally aging mice [3]. Thus, senescence is an irrefutable cause for aging and targeting them is warranted. But can they also be eliminated therapeutically? And are such methods then safe on their own? And last, but not least, would such methods be applicable to not merely delay, but also to reverse aging?

**Figure 1:**
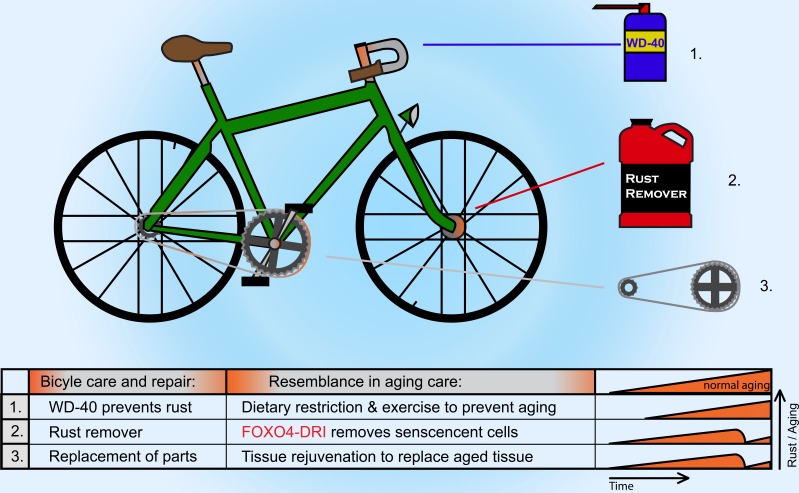
The aging cycle of life The analogy compares senescent cells in an aged body to rust on a racing bicycle. Different strategies can be used to prevent, treat and remove rust and aging. WD-40, a corrosion inhibitor, resembles dietary restriction and regular exercise to delay rust or aging. When rust and aging have already settled, the FOXO4-DRI peptide can act as a rust remover by inducing cell death in senescent cells. Last, the stimulation of tissue rejuvenation can promote a healthy, revitalized tissue that can be compared with the replacement of bicycle parts. The combination of these strategies may be complimentary in fighting aging and age-related defects.

## Le tour de FOXO. A demanding journey, but one with great rewards

A first surprise when trying to address these questions was that senescent cells recruit a factor called FOXO4 to sites of persistent DNA damage, structures absent in normal healthy cells [1]. This is intriguing as FOXO4 is considered to be the ugly little sister of FOXO1 and FOXO3, which do play major roles in processes ranging from stem cell function, differentiation, tumor suppression, and, aging [4]. In senescence, however, FOXO4 appears to act as a brake on the apoptosis response by sequestering p53. Prospective design of a D-Retro-Inversed Cell Penetrating Peptide that perturbs this interaction, named FOXO4-DRI, allowed for nuclear release of active p53, followed by cell-intrinsic apoptosis and selective elimination of the senescent cells.

Recent work elegantly proved that senescent cells are a major cause for the toxic side effects caused by multiple independent forms of chemotherapy [5]. Excitingly, FOXO4-DRI counteracted senescence caused by Doxorubicin and reversed liver toxicity providing evidence that therapeutic removal of senescent cells by FOXO4-DRI can counteract at least some aspects of chemotoxicity. Proceeding from this acute senescence-induction model, we then focused on fast aging *Xpd^TTD/TTD^* mice, which spontaneously develop senescence in an accelerated fashion, in parallel with organism-wide deterioration. FOXO4-DRI proved to significantly restore their health on multiple levels. Though not purposefully investigated, it was strikingly apparent that FOXO4-DRI treated mice regained fur and improved their voluntary exploratory behavior compared to PBS treated counterparts. In addition, kidney function markedly restored. Naturally aged mice showed more biological noise than the fast aging mice, making these features more difficult to address. But at least the effects on renal function were clearly prevalent in naturally aged mice. Thus, using FOXO4-DRI it indeed appears possible to not just delay aging but also reverse at least certain signs of it. So, what's next?

## A combination of efforts to best the mountains ahead

Aging is ultimately still inevitable. But perhaps it can be strongly postponed, or even reversed, when independent anti-aging therapies are combined? It remains to be determined whether extension of lifespan is possible in humans [6], let alone whether this is desirable and then to what age? After all, life could at some point not simply “complete”? While this might be true for some, nobody likes being sick and frail. Imagine the possibilities if we would be able to enjoy our time with loved ones, exercise and travel more and simply just enjoy life in good health, instead of spending it in a retirement home.

Extending the healthy years of life is now closer than ever, but we are still not there yet. While mechanics can remove defective parts from an old bicycle, it is far more challenging to remove damaged parts from an old body (Figure 1). Anti-aging strategies have therefore necessarily focused thus far on stalling the inevitable for as long as possible by eating less and exercising more. A multitude of new diets make it to the mainstream public each year, but ironically, people tend to exercise less and gain more and more weight. This argues that instead of focusing so much on dietary interventions, independent approaches deserve to be investigated. Here, we underscored the potential of therapeutic elimination of senescent cells, for instance by FOXO4-DRI. In addition, exciting developments were recently reported in the field of stem cell biology, where it was shown that transient expression of the Yamanaka stem cell factors can promote tissue rejuvenation [7]. This is not yet therapeutically applicable, but this will most likely only be a matter of time.

It is no longer merely science-fiction to restore healthspan with rationally designed approaches. To fully achieve the best possible outcome, it will therefore deserve special consideration to combine existing methods to delay aging with the recently developed therapies that counter senescence and promote tissue rejuvenation. With these, we finally have exciting tools to maintain and repair the aging cycle of life (Figure 1). Time to gear up and head for the finish!
